# Supplementary material for: Endorsement of gender stereotypes in gender diverse and cisgender adolescents and their parents
Source: PLoS One. 2022 Jun 14;17(6):e0269784. doi: 10.1371/journal.pone.0269784 (PMC9197027; doi:10.1371/journal.pone.0269784)
Supplement: S1 File — (DOCX) [file pone.0269784.s001.docx]

# Supporting Information

# Section 1: Stereotype endorsement as a binary variable

In addition to the analysis we presented in the main text in which we considered each response to be on a continuous 1-5 scale, we also analyzed participants’ responses as a binary variable using two different scoring methods. We conducted these analyses to more closely follow the guidance given by Liben & Bigler (2002) on how the OAT-AM should be scored.

***Scoring Method #1***

In the first analysis, we scored participants’ responses to indicate whether, on each item, participants indicated a stereotype-consistent response to a particular item (akin to choosing an answer scored as ‘4’ or ‘5’ in the scale described in the main text), or whether they chose a stereotype-inconsistent response (a ‘1’, ‘2’, or ‘3’). We then calculate the proportion of non-stereotypical responding for each parent and adolescent. We fit a mixed-effects generalized linear model with a logit linking function in which the dependent variable was each participant’s proportion of stereotype-inconsistent responses, and the predictors were the gender diversity of the adolescent (*cisgender* vs. *gender diverse*), the question set (*adolescent self-report* vs. *parent self-report*), and the interaction between these two predictors. Additionally, we included a random intercept for each family. Thus, this analysis matches the main analysis presented in the Results section, with the adjustment that the outcome variable is a proportion between 0 and 1, as opposed to a mean score. The final model specification was as follows: proportion of responses without stereotype endorsement ~ question set * gender diverse identification of adolescent + (1 | family).

The full regression output is shown in Table S1. As in the main analysis, there was a significant main effect of question set, such that *parent self-report* responses were more likely to not contain endorsement of gender stereotypes. Additionally, we see two effects not seen in the main analysis. First, we see a main effect of gender diversity, with participants in the *cisgender group* showing very slightly less stereotype endorsement than participants in the *gender diverse group*. Second, a significant interaction of gender diversity and question set emerged, such that the gap between levels of adolescents’ and parents’ stereotyping was smaller in the *gender diverse group* than in the *cisgender group*. In the *cisgender group*, the predicted proportion of non-stereotype endorsing responses on the *parent self-report* measure was 0.986, while the predicted proportion of non-stereotype endorsing responses on the *adolescent self-report* measure was 0.962. In the *gender diverse group*, the predicted proportion of non-stereotype endorsing responses on the *parent self-report* measure was 0.973, while the predicted proportion of non-stereotype endorsing responses on the *adolescent self-report* measure was 0.957. In sum, parents in both groups were more likely to choose non-stereotyped responses than their children, though the effect was very small and all groups are overwhelmingly likely to choose non-stereotyped responses.

***Scoring Method #2***

The second scoring method was very similar to Scoring Method #1 above; the only difference is that we coded items as showing no stereotype endorsement if they chose a response of ‘3’, indicating that “both men and women” should possess the trait in question, and showing stereotype endorsement if they chose any response other than 3. This scoring procedure closely aligns with the suggested scoring procedure in Liben & Bigler (2002).

As with Scoring Method #1, we fit a mixed-effects generalized linear model with a logit linking function in which the dependent measure was the proportion of responses in which participants did not endorse stereotypes (i.e., chose a value of ‘3’), and the predictors were the gender diversity of the adolescent (*cisgender* vs. *gender diverse),* the question set (*adolescent self-report* and *parent self-report*), and the interaction between these two predictors. We also included a random intercept of family. The model specification was the same as in Scoring Method #1, with the exception that the proportion of responses without stereotype endorsement in this model corresponds to the proportion of a participant’s responses in which they chose ‘3’.

The full regression output is in Table S2. The results are nearly identical to those obtained using Scoring Method #1. In the *cisgender group*, the predicted proportion of non-stereotype endorsing responses on the *parent self-report* measure was 0.989, while the predicted proportion of non-stereotype endorsing responses on the *adolescent self-report* measure was 0.965. In the *gender diverse group*, the predicted proportion of non-stereotype endorsing responses on the *parent self-report* measure was 0.972, while the predicted proportion of non-stereotype endorsing responses on the *adolescent self-report* measure was 0.951. In sum, as in the analysis using Scoring Method #1, parents in both groups were slightly more likely to choose non-stereotyped responses than their children, though the effect was very small and all groups are overwhelmingly likely to choose non-stereotyped responses.

**Table S1.** Regression output: Stereotype endorsement as a binary variable (Scoring Method 1). Reference group is cisgender adolescent self-report.

| Predictor | Estimate | Standard Error | z-value | *p*-value |
| --- | --- | --- | --- | --- |
| Intercept | 4.26 | 0.21 | 20.60 | *< .*001 |
| Parent self-report (vs. adolescent) | -1.03 | 0.11 | -9.02 | *< .*001 |
| Gender diverse group (vs. cisgender) | -0.69 | 0.27 | -2.56 | 0.01 |
| Parent self-report * gender diverse group | 0.56 | 0.15 | 3.66 | *< .*001 |

**Table S2.** Regression output: Stereotype endorsement as a binary variable (Scoring Method 2). Reference group is cisgender adolescent self-report.

| Predictor | Estimate | Standard Error | *z*-value | *p*-value |
| --- | --- | --- | --- | --- |
| Intercept | 4.48 | 0.23 | 19.10 | *< .*001 |
| Parent self-report (vs. adolescent) | -1.18 | 0.11 | -10.23 | *< .*001 |
| Gender diverse group (vs. cisgender) | -0.93 | 0.31 | -3.02 | *< .*001 |
| Parent self-report * gender diverse group | 0.60 | 0.15 | 3.89 | *< .*001 |

# Section 2: Bayes Factor Analysis

As an exploratory analysis, we also computed Bayes Factors in order to quantify, given the data observed, the evidence in favor of a null hypothesis (i.e., that the population difference in mean scores between the *gender diverse* and *cisgender groups* is zero) over an alternative hypothesis (that the population difference in mean scores is not zero) for each of the three measures assessed (*adolescent self-report*, *adolescent predictions about the parent*, and *parent self-report*). We first averaged scores within participants and measures, so that adolescents each had one mean score on the *adolescent self-report* measure and the *adolescent predictions about the parent* measure, and parents each had one mean score on the *parent self-report* measure. We then used the BayesFactor package in R (Morey et al., 2015) to calculate the ratio between the marginal likelihoods of the null and alternative hypotheses, where the prior distribution of the standardized mean difference between groups varies according to a Cauchy distribution with scale parameter $\sqrt{1/2}$ under the alternative hypothesis, and the standardized mean difference between groups under the null hypothesis is assumed to be 0. On the *adolescent self-report* scale, we obtained a Bayes Factor of 0.1364, indicating that the observed data were 7.33 times more likely under the null hypothesis than the alternative. On the *adolescent predictions about the parent* measure, we obtained a Bayes Factor of 0.1256, indicating that the observed data were 7.964 times more likely under the null hypothesis than under the alternative. Finally, on the *parent self-report* measures, we obtained a Bayes Factor of 0.1802, indicating that the observed data were 5.551 times more likely under the null hypothesis than under the alternative.

# Section 3: Additional demographic tables

**Table S3**: Detailed breakdown of gender identities in parent sample.



| Category | Parents: Gender Diverse Group | Parents: Cisgender Group Woman |
| --- | --- | --- |
| Woman | 118 | 155 |
| Man | 19 | 4 |
| Nonbinary | 0 | 1 |
| Gender fluid | 2 | 0 |
| Transmasculine | 1 | 0 |
| No gender reported | 3 | 0 |

**Table S4**. Detailed breakdown of gender identities in adolescent sample. All entries below "agender" were written in by adolescents (as opposed to chosen from a drop-down menu).

| Category | Adolescents: Gender diverse group | Adolescents:  Cisgender group |
| --- | --- | --- |
| Girl | 49 | 92 |
| Boy | 60 | 81 |
| Nonbinary | 18 | 0 |
| Gender fluid | 6 | 0 |
| Agender | 5 | 0 |
| demi-girl, some where between girl and nonbinary | 1 | 0 |
| demi-guy (mostly male, but partially agender) | 1 | 0 |
| Gender nonconforming | 1 | 0 |
| I don’t identify as it, I simply am female | 1 | 0 |
| nonbinary/boy | 1 | 0 |
| Trans masc nonbinary | 1 | 0 |
| Attack Helicopter | 0 | 1 |

# Section 4: Potential group differences in correlation between parent and adolescent measures

We conducted exploratory analyses that further investigate the relationships between adolescents’ responses to the OAT-AM and their parents’ responses. To do so, we first calculated mean scores on each of the three measures (*parent self-report*, *adolescent prediction about the parent*, and *adolescent prediction about the parent*) for each parent-adolescent dyad. We then fit two mixed-effects linear regression models.

The first regression (Table S5) predicted scores on the *adolescent prediction about the parent* measure as a function of 3 predictors: scores on the *parent self-report* measure, whether the adolescent was *gender diverse* or *cisgender*, and the interaction between these two factors; there was also a random intercept of family, to account for the fact that some families are represented multiple times in the dataset. This analysis explores the question: were adolescents’ predictions about their parents actually predictive of their parents’ stereotyping, and did this predictiveness differ between *gender diverse* and *cisgender* adolescents?

This model failed to show a main effect of *parent self-report* as a predictor for the *adolescent prediction about the caregiver* (*β* = -0.11, *p* = 0.55), but did show a significant main effect of the gender diversity of the adolescent (*gender diverse* vs. *cisgender*; *β*_gender diverse_ = -1.51, *p* = 0.03), as well as a significant interaction between the two aforementioned predictors (*β* = 0.50, *p* = 0.03). However, given the exploratory nature of this analysis, we encourage caution in interpreting these effects.

The second regression (Table S6) predicted scores on the *adolescent self-report* measure as a function of 3 predictors: scores on the *parent self-report* measure, whether the adolescent was *gender diverse* or *cisgender*, and the interaction between these two factors. This analysis explores the question: were adolescents’ own stereotyping scores predictive of their parents’ stereotyping, and did this predictiveness differ between *gender diverse* and *cisgender* adolescents?

This model^[[1]](#footnote-1)^ failed to show significant a main effect of *parent self-report* score, indicating that *parent self-report* scores were not a significant predictor of *adolescent self-report* scores (*β* = 0.09, *p* = 0.57); additionally, there was no main effect of the adolescent’s gender diversity (*gender diverse* vs. *cisgender*; *β* = -0.65, *p* = 0.29), nor was there a significant interaction between the two aforementioned predictors (*β* = 0.21, *p* = 0.30).

**Table S5**. Results from linear regression predicting *parents’ self-report* scores from the *adolescent prediction about the parent*, the gender diversity of the adolescent in the dyad, and the interaction between these predictors.

| Predictor | Estimate | Standard Error | df | *t-*value | *p-*value |
| --- | --- | --- | --- | --- | --- |
| Intercept | 3.46 | 0.58 | 284.40 | 5.94 | *< .*001 |
| Parent self-report score | -0.11 | 0.19 | 284.25 | -0.60 | 0.55 |
| Gender diverse group (vs. cisgender) | -1.51 | 0.71 | 287.63 | -2.12 | 0.03 |
| Gender diverse group * Parent self-report measure | 0.50 | 0.23 | 287.53 | 2.12 | 0.03 |

**Table S6**. Results from linear regression predicting *parents’ self-report* scores from the *adolescent self-report measure*, the gender diversity of the adolescent in the dyad, and the interaction between these predictors.

| Predictor | Estimate | Standard Error | df | *t*-value | *p*-value |
| --- | --- | --- | --- | --- | --- |
| Intercept | 2.82 | 0.49 | 314 | 5.70 | *< .*001 |
| Parent self-report score | 0.09 | 0.16 | 314 | 0.56 | 0.57 |
| Gender diverse group (vs. cisgender) | -0.65 | 0.61 | 314 | -1.06 | 0.29 |
| Gender diverse group * Parent self-report measure | 0.21 | 0.20 | 314 | 1.04 | 0.30 |

# Section 5: Differences in endorsement of masculine and feminine stereotypes.

# As an exploratory analysis, we examined whether adolescents and their parents showed differing levels of stereotype endorsement for masculine versus feminine stereotypes.

# First, collapsing across families with gender diverse and cisgender adolescents, we fit two linear mixed-effects regression models predicting adolescents’ and parents’ stereotype endorsement from a fixed effect of stereotype gender (masculine or feminine) and a random intercept of subject. Both adolescents (*β*_masculine_ = -0.07, *p* = .002; full regression output in Table S7) and parents^[[2]](#footnote-2)^ (*β*_masculine_ = -0.05, *p* < .001; full regression output in Table S8) showed less stereotype endorsement of masculine stereotypes than feminine stereotypes in these models. We then fit two additional linear mixed-effects regression models predicting adolescents’ and parents’^[[3]](#footnote-3)^ stereotype endorsement with fixed effects of stereotype gender (masculine or feminine), the gender diversity of the adolescent (*gender diverse* or *cisgender*), and the interaction between these two factors, as well as a random intercept of subject; neither of these regressions showed significant main effects of stereotype gender (*β*_adolescents; masculine_ = -0.04, *p* = 0.16; *β*_parents; masculine_ = -0.03, *p* = 0.21; full regression output in Tables S9 and S10), and there were no interactions with the gender diversity of the adolescent. Thus, slightly higher stereotype endorsement for feminine items was observed among adolescents and parents, but neither group showed a significant effect when including the adolescent’s gender diversity as a predictor in the regression.

**Table S7**: Results for linear regression predicting *adolescent self-report* scores from the gender of the stereotype in each item (masculine or feminine).

| Predictor | Estimate | Standard Error | df | *t-*value | *p-*value |
| --- | --- | --- | --- | --- | --- |
| Intercept | 3.13 | 0.01 | 631.03 | 209.11 | < .001 |
| Stereotype gender: Masculine | -0.07 | 0.02 | 317.11 | -3.17 | .002 |

**Table S8**: Results for linear regression predicting *parent self-report* scores from the gender of the stereotype in each item (masculine or feminine).

| Predictor | Estimate | Standard Error | df | *t-*value | *p-*value |
| --- | --- | --- | --- | --- | --- |
| Intercept | 3.07 | 0.01 | 603 | 285.34 | < .001 |
| Stereotype gender: Masculine | -0.05 | 0.02 | 603 | -3.43 | < .001 |

**Table S9**: Results for linear regression predicting *adolescent self-report* scores from the gender of the stereotype in each item (masculine or feminine), the gender diversity of the adolescent (*gender diverse* or *cisgender*), and the interaction between these two factors.

| Predictor | Estimate | Standard Error | df | *t-*value | *p-*value |
| --- | --- | --- | --- | --- | --- |
| Intercept | 3.12 | 0.02 | 628.82 | 154.25 | < .001 |
| Stereotype gender: Masculine | -0.04 | 0.03 | 315.59 | -1.40 | 0.16 |
| Gender diverse group (vs. cisgender) | 0.02 | 0.03 | 628.82 | 0.65 | 0.52 |
| Stereotype gender: Masculine * Gender diverse group | -0.06 | 0.04 | 316.19 | -1.41 | 0.16 |

**Table S10**: Results for linear regression predicting *parent self-report* scores from the gender of the stereotype in each item (masculine or feminine), the gender diversity of the parent’s adolescent (*gender diverse* or *cisgender*), and the interaction between these two factors.

| Predictor | Estimate | Standard Error | df | *t-*value | *p-*value |
| --- | --- | --- | --- | --- | --- |
| Intercept | 3.06 | 0.01 | 601 | 206.43 | < .001 |
| Stereotype gender: Masculine | -0.03 | 0.02 | 601 | -1.26 | 0.21 |
| Gender diverse group (vs. cisgender) | 0.04 | 0.02 | 601 | 1.75 | 0.08 |
| Stereotype gender: Masculine * Gender diverse group | -0.05 | 0.03 | 601 | -1.79 | 0.07 |

# Section 6: Differences in endorsement of personality-related vs. academic/extracurricular stereotypes.

# As another exploratory analysis, we examined whether adolescents and their parents showed differing levels of stereotype endorsement for academic/extracurricular- versus personality- related stereotypes.

# First, collapsing across families with gender diverse and cisgender adolescents, we fit two linear mixed-effects regression models predicting adolescents’ and parents’ stereotype endorsement from a fixed effect of stereotype domain (personality or academic/extracurricular) and a random intercept of subject. Both adolescents (*β* = 0.09, *p* < .001; full regression output in Table S11) and parents^[[4]](#footnote-4)^ (*β* = 0.06, *p* < .001; full regression output in Table S12) showed greater stereotype endorsement of personality-related stereotypes than academic/extracurricular stereotypes in these models. We then fit two additional linear mixed-effects regression models predicting adolescents’ and parents’^[[5]](#footnote-5)^ stereotype endorsement with fixed effects of stereotype domain (personality or academic/extracurricular), the gender diversity of the adolescent (*gender diverse* or *cisgender*), and the interaction between these two factors, as well as a random intercept of subject. Both of these regressions showed significant main effects of stereotype domain (*β*_adolescents; personality_ = 0.08, *p* < .001; *β*_parents; personality_ = 0.06, *p* < .001; full regression output in Tables S13 and S14). Thus, slightly higher stereotype endorsement for feminine items was observed among adolescents and parents.

**Table S11**: Results for linear regression predicting *adolescent self-report* scores from the domain of the stereotype in each item (personality or academic/extracurricular).

| Predictor | Estimate | Standard Error | df | *t-*value | *p-*value |
| --- | --- | --- | --- | --- | --- |
| Intercept | 3.04 | 0.01 | 544.18 | 242.34 | < .001 |
| Stereotype gender: Personality (vs. academic/extracurricular) | 0.09 | 0.01 | 316.69 | 6.31 | < .001 |

**Table S12**: Results for linear regression predicting *parent self-report* scores from the domain of the stereotype in each item (personality or academic/extracurricular).

| Predictor | Estimate | Standard Error | df | *t-*value | *p-*value |
| --- | --- | --- | --- | --- | --- |
| Intercept | 3.01 | 0.01 | 601 | 337.91 | < .001 |
| Stereotype gender: Personality (vs. academic/extracurricular) | 0.06 | 0.01 | 601 | 4.89 | < .001 |

**Table S13**: Results for linear regression predicting *adolescent self-report* scores from the domain of the stereotype in each item (personality or academic/extracurricular), the gender diversity of the adolescent (*gender diverse* or *cisgender*), and the interaction between these two factors.

| Predictor | Estimate | Standard Error | df | *t-*value | *p-*value |
| --- | --- | --- | --- | --- | --- |
| Intercept | 3.05 | 0.02 | 541.75 | 179.58 | < .001 |
| Stereotype gender: Personality (vs. academic/extracurricular) | 0.08 | 0.02 | 314.87 | 4.55 | < .001 |
| Gender diverse group (vs. cisgender) | -0.01 | 0.03 | 542.72 | -0.49 | 0.63 |
| Stereotype gender: Personality * Gender diverse group | 0.00 | 0.03 | 315.89 | 0.17 | 0.86 |

**Table S14**: Results for linear regression predicting *parent self-report* scores from the domain of the stereotype in each item (personality or academic/extracurricular), the gender diversity of the adolescent (*gender diverse* or *cisgender*), and the interaction between these two factors.

| Predictor | Estimate | Standard Error | df | *t-*value | *p-*value |
| --- | --- | --- | --- | --- | --- |
| Intercept | 3.00 | 0.01 | 599 | 245.28 | < .001 |
| Stereotype gender: Personality (vs. academic/extracurricular) | 0.06 | 0.02 | 599 | 3.61 | < .001 |
| Gender diverse group (vs. cisgender) | 0.01 | 0.02 | 599 | 0.73 | 0.47 |
| Stereotype gender: Personality * Gender diverse group | 0.00 | 0.03 | 599 | -0.08 | 0.94 |

# Section 7: Correlations between stereotyping measures.

|  | Adolescent self-report | Adolescent prediction about the parent | Parent self-report |
| --- | --- | --- | --- |
| Adolescent self-report | 1 |  |  |
| Adolescent prediction about the parent | 0.251 | 1 |  |
| Parent self-report | 0.133 | 0.109 | 1 |

**Table S15:** Pearson’s *r* correlations between stereotype measures. (*Note:* In calculating these Pearson’s *r* values, some *parent self-report* values are duplicated since some parents had multiple adolescents participate in the study.)

## References

Liben, L. S., & Bigler, R. S. (2002). The developmental course of gender differentiation: Conceptualizing, measuring, and evaluating constructs and pathways. Monographs of the Society for Research in Child Development, 67(2), vii–147. <https://doi.org/10.1111/1540-5834.t01-1-00187>

Morey, R. D., Rouder, J. N., Jamil, T., Urbanek, S., Forner, K., & Ly, A. (2015). Package ‘bayesfactor’. <http://cran/r-projectorg/web/packages/BayesFactor/BayesFactor.pdf>

1. This model obtained a singular fit; however, since regression coefficients were almost identical without the random intercepts, we report the mixed-effect model results here. [↑](#footnote-ref-1)
2. This model obtained a singular fit; however, since regression coefficients were almost identical without the random intercepts, we report the mixed-effect model results here. [↑](#footnote-ref-2)
3. This model obtained a singular fit; however, since regression coefficients were almost identical without the random intercepts, we report the mixed-effect model results here. [↑](#footnote-ref-3)
4. This model obtained a singular fit; however, since regression coefficients were almost identical without the random intercepts, we report the mixed-effect model results here. [↑](#footnote-ref-4)
5. This model obtained a singular fit; however, since regression coefficients were almost identical without the random intercepts, we report the mixed-effect model results here. [↑](#footnote-ref-5)
