# Supplementary material for: Endorsement of gender stereotypes in gender diverse and cisgender adolescents and their parents
Source: PLoS One. 2022 Jun 14;17(6):e0269784. doi: 10.1371/journal.pone.0269784 (PMC9197027; doi:10.1371/journal.pone.0269784)
Supplement: Attachment — Submitted filename: response letter (deMayo, Kahn-Samuelson, & Olson).docx [file pone.0269784.s002.docx]

Dear Dr. Steele,

Thank you so much for extending us the opportunity to resubmit our manuscript, “Endorsement of gender stereotypes in gender diverse and cisgender adolescents and their parents” and for giving us some extra time to complete the revision. We are pleased to submit a revised version of the manuscript.

Overall, the most notable changes to the manuscript include:

- We more clearly state our research questions and better match the analyses presented with those research questions.
- We provide more relevant background on the research questions.
- We have broadened the analyses reported by including exploratory analyses examining gender stereotype endorsement as a function of parent and adolescent gender.
- We reformatted the paper into APA style.
- We clarify that we only used the trait subscale of the OAT-AM, not the entire measure as it was originally published.
- Finally, as part of a lab-wide effort to increase transparency and consistency in how we exclude and report participants across papers, we added justification of the exact reasons why participants were dropped to ensure consistency with other papers . As a result, the sample size differs very slightly (N=5 fewer *gender diverse* parent-teen dyads, N = 3 fewer *cisgender* parent-teen dyads), a change that resulted in no differences in the results. We are now clearer in labeling the reasons why each excluded participant was dropped.

Below, you will find a point-by-point response to comments made in the first round of review.

Thank you again for your flexibility and we look forward to hearing your decision.

Sincerely,

Benny deMayo

Shira Kahn-Samuelson

Kristina Olson

*Please be sure to submit the revised manuscript in the appropriate format (https://journals.plos.org/plosone/s/submission-guidelines). As one example “Manuscript text should be double-spaced. Do not format text in multiple columns.” You might want to refer to the APA manual (7th edition) for suggestions as well.*

Thank you, we have made sure the paper is appropriately formatted.

*On page two, you indicate that several recent studies have examined gender stereotyping in transgender youth, but then proceed to review papers with children. Please change the wording to reflect this. The more important question, raised also by Reviewer 1, is whether each of these studies were conducted with the same children. That is, are conclusions drawn about the gender stereotyping of gender diverse preschoolers, young children, and older children all using the same (or many of the same) participants as this study that is examining adolescents? If so, this should be clearly explained in the paper. Related to this, please clarify how many gender diverse adolescents were recruited from other sources.*

Thank you for this suggestion. We have made the language more consistent to reflect the fact that prior research has been conducted with children under the age of 11, but that our study involves adolescents between the ages of 13 and 17. We have also clarified how many participants in the present work are included in previous studies (n=8), and have specified how many gender diverse adolescents were recruited from other sources (n=64).

*Given that the OAT-AM is almost 20 year old, it seems possible that some of the items are dated. It would be useful to know more about this measure and the results. Was most of the variability driven by some items (if so, which ones) and did some items not vary at all? It would be useful if the items could be detailed on OSF either in the data file (e.g., Parent_OAT_1_Math) and/or in the variable explanations. In addition, it would be ideal to provide not only the final data file, but also a data file that includes all participants (prior to exclusions) on OSF.*

Thank you for this suggestion. We have included a table in the Supplementary Material that shows the mean score for each item on the scale and its standard error. We have also included reliability analysis for the items on the trait subscale of the OAT-AM in response to a comment from R2. Finally, we have included explanations of each of the items on the trait subscale of the OAT-AM on OSF (wording of items as presented to participants can be found in `oat_items.csv`), as well as versions of the data file with participants prior to exclusions (titled `kid_data_full_osf.csv` for the parents and `parent_data_full_osf.csv`). All of these files can be found in the `resubmission` folder in the OSF page: https://osf.io/yxs3r/files/

*In the final sentence of the abstract, you indicate that “these results suggest…” but none of the results reported to that point suggest what follows. This relates to your research questions and hypotheses – it does seem that one of the questions that you address is whether there will be explicit stereotype endorsement and not just a difference between the groups, and you might consider adding this as a research question when revising, as well as revising the abstract to address this inconsistency.*

Thank you for this suggestion. We have added another research question and a corresponding subsection in the “Results” section that more directly probes whether there was explicit stereotype endorsement at all, not just a difference between the groups. We have also accordingly edited the abstract to reflect this change.

*The analyses in the supplement did not make sense to me, as participants’ responses were recoded based on whether they selected 3 (no gender difference) or not. However, the direction of that ‘not’ is important, as it reflects gender stereotyping, or counterstereotypical beliefs. I would recommend re-running these analyses, pitting stereotype-consistent responses against non-stereotype-consistent responses (e.g., 3 or counterstereotypical). Given the restricted variability this might not make any meaningful difference in the results, but this seems a more appropriate analytic approach given your research questions.*

Thank you for this recommendation. We have re-run this analysis in the supplementary material with the gender stereotyping variable recoded as you suggest. We agree with you that knowing the direction matters. In addition, we retained the initial coding (based on whether they selected 3 or not) in the supplement as well, since this is the method of response scoring suggested by the original creators of the OAT-AM, and we want interested readers to be able to compare our results to that work.

*My final point is that it would be ideal to expand the scope of this paper if possible. I wonder if there are additional questions that might be addressed, for example if some of these adolescents did provide responses at an earlier time that could be compared. Reviewer 2 provides several other suggestions using the current data (see points 6 & 7) or with additional data (see point 12). Given that the main finding is a lack of difference between groups combined with low variability and a general lack of stereotyping on an older measure, I believe that the findings would be more compelling with additional analyses, or ideally additional data.*

Thank you for this suggestion. We agree that additional data would make the findings more compelling, and we looked into the possibility of correlating past data from the same participants with the stereotyping scores reported here; however, only 8 of the adolescents in this study had completed a stereotyping measure in the past . Thus, there was not enough longitudinal data on the present construct to make an interesting addition to the paper.

Further, there are two other issues with adding more data from past administrations with this cohort. First, there is almost no variability in the present results meaning that it would be nearly impossible to observe any meaningful association between these results and anything else. If we move to an even further construct (e.g., peer preferences, self-esteem), we’d be even less likely to find an association. Second, many of the participants in the present work had never participated in a study with us before and therefore we would need to exclude them from such analyses, lowering our sample size, and further reducing the odds of finding significant associations over time.

We did, however, add some additional analyses of the present data in response to points 6 & 7 made by Reviewer 2 (examining gender stereotype endorsement by parents’ gender and adolescents’ gender); we also include an exploratory analysis in the Supplementary Materials that examines whether gender diverse adolescents’ stereotyping was more (or less) predictive of their parents’ stereotyping than cisgender adolescents’ stereotyping.

**Reviewer 1:**

*Reviewer #1: One concern about dual publication is the use of the same gender diverse adolescents across multiple manuscripts. I know this is a large data set and it is reasonable that it will result in multiple manuscripts, but given how little research has been conducted with gender diverse youth, the repeated publication from one data set may skew the knowledge base. I recommend citing all of the other publications using the same youth in the Participants section.*

*Otherwise, the analyses are appropriate and the text is clear and complete. The conclusions are appropriate based on the results.*

Thank you for this suggestion. We have cited all the other publications using the same youth in the participants section. Importantly, only 8 of the transgender participants completed a past stereotyping measure. Sixty-four of the gender diverse participants and 107 of the cisgender participants have never completed any studies with our team.

**Reviewer 2:**

*1. The Introduction could benefit from providing more of a theoretical framework, or at least providing more context to support hypotheses/predictions about why trans, compared with cis, adolescents (and their respective parents) would show more, less, or similar patterns of gender stereotyping. The authors should also give more background on parental gender socialization and associations between parent and adolescent gender stereotyping. Would the authors predict a difference in the strength of the association between trans vs. cis parent-adolescent dyads? If memory serves, Olson and Enright’s 2018 Dev Sci article reported that both trans children and their siblings were similarly accepting of gender nonconformity (compared with other cisgender controls). Could this reflect some differential parental socialization taking place with regard to acceptability of deviations from gender norms? There is also no explanation regarding the value of considering both parents’ actual gender stereotyping vs. adolescents’ perceptions of the extent to which their parents would gender stereotype. In short, the Introduction should be developed further to convey the theoretical and/or practical importance of the kinds of questions being asked here. To the extent that directional predictions (even if competing) can be offered, I encourage that as well.*

Thank you for this suggestion. We have scaffolded the introduction with more relevant discussion of past work that speaks to the inclusion of these measures and research questions. In particular, we have added more background on parental gender socialization as it informs hypotheses about whether parents of transgender or cisgender adolescents might show more or less explicit stereotype endorsement. In addition, we have added text that clarifies why we measured adolescents’ perceptions of the extent to which their parents would endorse gender stereotypes. While we did not have *a priori* directional predictions, we provide information about why one might speculate different patterns of results given prior literature.

*2. I found that there were several statements of fact that were not backed up by a citation. Examples include claims that the numbers of trans adolescents are increasing and that past work generally finds that parents influence their children’s thinking about gender (regarding this latter one, I wonder whether the authors meant children and adolescents because the latter seems more relevant to concentrate on here). I suggest the authors make sure to back up statements of fact with appropriate citations.*

Thank you for this suggestion; we have made sure that all broad statements in the article are supported by prior literature.

*3. What is the benefit of retaining participants who skipped all questions? Seems that if they did not provide data on the dependent variables, they should be dropped because they are leading to a skewed sense of the characteristics of the groups of participants who contributed the key data.*

We apologize for the error in reporting. Participants who completed no items were not included in analyses. (However, adolescents who completed the self-report items but none of the items predicting the parent’s stereotyping were included in the applicable analyses.)

*4. The samples do not seem to be matched on demographics. The cisgender sample appears to be more affluent and less white/more multiracial (based on the parent data). Are the authors concerned that this might be an important confound in their analyses? There is some research suggesting racial differences in gender development exist (e.g., work by May Ling Halim). Also, the cisgender controls were recruited from a participant database at the authors’ institution. In the present case, I assume this means that participants were from a relatively urban center in the US Pacific Northwest, which probably has a particular social climate. I wonder whether the gender diverse sample is from a more varied set of backgrounds given they were recruited from across the US and Canada, and whether this is also a relevant confound to consider in weighing the comparability of the participant groups.*

Thank you for bringing up this point. The samples are not perfectly matched on demographics. We have included chi-squared tests and two-sample t-tests and results in the participant demographics table to illustrate differences between the two groups. Nonetheless, given the general lack of difference on the dependent measures between the groups, we think it is unlikely that demographic differences between the two groups played a large role. However, we have added text to the limitations section highlighting that the two groups are not matched on demographics and that the biases, particularly in the cisgender group, might explain why we observed so little stereotyping.

*5. Can the authors please say more about the recruitment method for online participants as well as for the other samples? I know this is part of a larger project and the authors have maybe shared some of these details elsewhere (especially on the longitudinal sample), but it’s not clear for those who are maybe only reading this paper from this team. Also, I wonder how this sample relates to prior ones that this group reported on with respect to gender stereotyping. There were three relevant studies from this team that were reviewed in the Introduction. Were the participants in this study the same as any of those prior ones? Or is this a completely different cohort?*

Thank you for this suggestion. We have included text that clarifies (a) how participants from the Trans Youth Project cohort were recruited (n=79), (b) how many of the gender diverse children in this paper have been profiled in prior studies (n=8), and (c) how gender diverse participants who were *not* part of the Trans Youth Project cohort were recruited (n = 64).

*6. The authors do not note the parent gender. There is literature suggesting mothers and fathers hold different attitudes about gender roles/stereotypes. I suggest reviewing that literature and analyzing by parent gender. Perhaps see work by Joyce Endendijk and colleagues on this topic.*

Thank you for this suggestion. We have included text in the Results section which indicates how many parents of each gender there were in each group, as well as an exploratory analysis examining whether there were differences in stereotyping between parents who identified as men and parents who identified as women. We have also included text citing literature about how mothers and fathers hold different attitudes about gender roles and stereotypes, including work by Endendijk.

*7. The authors do not note the adolescent participants’ gender breakdown. Are there adolescent gender differences among cis samples in gender stereotyping? Any reason to suspect there might be differences between trans boys vs. trans girls vs. nonbinary, and so on? Even a preliminary analysis of this question would be important/interesting. In any case, it is presently unclear how comparable the samples are with respect to the gender composition with regard to cis/trans feminine/masculine individuals.*

Thank you for this suggestion. We have included information about how many adolescents of each gender there were in each group (Table 2) and the means and standard errors on the stereotyping measure for adolescents of different genders (Table 5). Further, we have included an ANOVA that tests for differences in stereotyping by gender.

*8. Can the authors please provide reliability analysis data on the OAT-AM for the present sample? Are all the items on this scale contributing to reliability? For example, there is some recent work suggesting that the stereotype that boys are superior at math is not always endorsed in adolescent samples (Morrissey et al 2019 in J Adolescence).*

Thank you for this suggestion. In the results section, we have included Crohnbach’s alpha values in the results for each of the three iterations of the trait subscale of the OAT-AM that we administered. We do not include the full analysis showing how much item contributed to reliability; there were no items that brought down reliability of the scale more than .03. We do note, however, that reliability on the parent self-report measure is lower than the others, and discuss potential reasons for the low variability.

*9. The authors state that the OAT-AM used in the current study was adapted lightly. Please explain.*

We have clarified that the trait subscale of the OAT-AM was adapted by removing the “Neither men nor women” option and adding a “skip” option for each item.

*10. It would be helpful to explain how the OAT-AM was scored in the Method section. A statistical analysis subsection would also be helpful to evaluate/understand the analytic approach. As is, the research questions and analyses are all somewhat vague, which makes it difficult to discern whether the optimal approach is being employed.*

Thank you for this suggestion. We have clarified in the Methods section how the OAT-AM is scored. Additionally, we have revised the results section around 3 questions, which are hopefully more clear than they were in the first submission.

- The first research question is whether participants showed gender stereotyping at all, without considering group differences. To answer this question, we include a one-sample t-test to test whether the mean value across all participants in the gender-stereotyping measure is different from 3 (the response which corresponds to ‘no gender stereotyping’).
- The second research question is whether there are group differences (both cisgender vs. gender diverse, as well as adolescents vs. parents) in gender stereotyping endorsement. To answer this question, we fit a mixed-effects linear regression predicting an individual’s mean stereotyping score from (1) whether they are an adolescent or a parent and (2) whether they are from a family with a cisgender teen participant or a family with a gender diverse teen participant.
- The third research question is whether adolescents’ gender stereotype endorsement, and their assumptions about their caregivers’ gender stereotype endorsement are predictive of parents’ gender stereotype endorsement. To that effect, we conduct linear regressions that examine the strength of the relationship between adolescents’ responses (of both types) and their parents’ responses.

*11. Tables 3: Please provide a more descriptive title.*

We have provided more descriptive titles.

*12. A main finding is that participants, regardless of group or age, were unlikely to endorse prescriptive gender stereotypes. One wonders what might have happened had the authors measured descriptive stereotypes. I also wonder whether we are now in an era where people hold (or at least report) explicit views that run contrary to traditional gender stereotypes. Perhaps an implicit measure would yield some different results. Given this team’s expertise in this area, I would be interested to see some discussion of these possibilities folded into this paper.*

Thank you for this suggestion. We have included text in the discussion section that suggests these areas as future topics of interest. We agree that they may be more productive than this focus on prescriptive work moving forward (though we didn’t know that before we found these results.)

*13. Page 8, line 255: The authors are making the point that one study found trans children ages 6-8 years gender stereotyped less. But in the Introduction they noted that trans children that study were similar to their cis siblings. So, it seems a little dubious to me to claim in the Discussion that the study of 6-8 year-olds is finding something that suggests a trans vs. cis difference.*

Great point. We have amended the language to indicate that trans youth and their siblings differed from unrelated cisgender youth in their stereotyping.

*14. The figure quality appeared “fuzzy” on my end. Consider revising to higher resolution.*

Thank you for this suggestion. We have made sure the figure appears in higher resolution.
