# Supplementary material for: Endorsement of gender stereotypes in gender diverse and cisgender adolescents and their parents
Source: PLoS One. 2022 Jun 14;17(6):e0269784. doi: 10.1371/journal.pone.0269784 (PMC9197027; doi:10.1371/journal.pone.0269784)
Supplement: Attachment — Submitted filename: response letter.docx [file pone.0269784.s003.docx]

Dear Dr. Steele,

Thank you so much for allowing us to revise and resubmit our manuscript, “Endorsement of gender stereotypes in gender diverse and cisgender adolescents and their parents”.

The most notable changes to the manuscript include:

- We have added analyses probing whether (a) masculine and feminine stereotypes are endorsed to differing extents and (b) whether stereotypes in different domains - academic vs. personality - are endorsed to differing extents. Specifically, as we describe below, we include tables in the manuscript that show means and standard deviations of stereotyping scores broken down on both of these dimensions, and refer readers to the supplementary materials for more detailed statistical inference probing effects related to stereotype gender and domain.
- We have added more information about the measure - most notably, a table with all of the items and their means and standard errors - into the manuscript itself.

Below, you will find a point-by-point response to comments from your most recent review of this manuscript.

Thank you again so much for your time in reviewing this work and allowing us to resubmit. We look forward to hearing your decision.

Sincerely,

Benjamin deMayo

Shira Kahn-Samuelson

Kristina Olson

*My main concern remains whether this provides sufficiently original research to warrant publication in this journal. I believe that it does, owing largely to the need for additional literature examining the social cognition of transgender youth. This also includes stereotyping by both youth and their parents, as well as youth’s expectations around their parents’ results. This is impressive. One additional question that is not addressed, and that I believe could help to bolster the novelty of this research, would be to also examine male and female stereotypes separately. That is, are there differences for feminine stereotypes or masculine stereotypes? Would you expect differences between these groups on either? This of course would need to be noted as a post-hoc analysis but might add to the substance of the article and possible the findings (e.g., in the case where no differences emerge). Similarly, on page 14 you suggest that more stereotyping occurred for personality traits versus academic domains. Additional analyses could examine this statistically as a post-hoc analysis (although you would need to note that should be interpreted with caution due to the post-hoc nature of this type of analysis). One or both of these might be most appropriately added to the supplement as opposed to the main text and I will leave that to you to decide. In short, I felt that some interesting questions were left unaddressed and addressing them would help to contribute the concerns previously raised by Reviewer 2 regarding the contribution of this article.*

**Thank you for these suggestions. Regarding originality, a few aspects of this work that we believe are an original contribution are: this is the first work on gender stereotyping in transgender adolescents (that we know of!), this is a contemporary test of a “classic” and common measure – that perhaps suggests the measure is getting to be less useful (thereby suggesting researchers might opt for others in the future), and this work is original in its inclusion of teens and their parents to look for within family associations.**

**In addition, we agree that the suggested analyses further increase the novelty of the work. In the “Gender stereotype endorsement” section of the Results (page 17), we include tables showing the means and standard errors for stereotyping scores, broken down both by the gender of the stereotypes in the items (masculine vs. feminine) and the domain of the stereotype (academic- vs. personality- related). We note that mean scores are higher for personality-related and feminine stereotypes, and point readers to a more detailed analysis examining these effects in the supplement (S5 and S6).**

**As you noted, we did not preregister a specific prediction for differential endorsement of masculine vs. feminine stereotypes, though some literature suggests that people are generally more comfortable with girls showing masculine stereotypes than the reverse (e.g., Martin, 1990; Coyle, Fulcher, & Trubutschek, 2016), which is consistent with our finding that people endorsed feminine stereotypes as being more for girls (only) than masculine stereotypes were for boys (only).**

*I was a bit confused by some of the information in Table 1. For one, I believe the chi-squared tests are comparing two groups (and show difference between (not among) groups?). For gender, I assume this is between woman and men with other/not reported being excluded from the analysis? For yearly income, it was not clear to me what was being compared. Please clarify.*

**Thank you for this suggestion; we have clarified what is being compared in each of the tests in Table 1. Specifically, we added notes that specify what is being compared in each of the chi-squared tests in both Table 1 and Table 2. For the chi-square tests comparing race, we are comparing white or non-white groups (because the small cells of specific groups violate assumptions of the chi-square analyses). For the gender comparison, we bin men together with “other/not reported” since participant N’s for men and “other/not reported” are so low. For the yearly income comparison, we converted participants’ reported income categories to a 1-5 scale (1 being the lowest, 5 being the highest) and ran a *t*-test comparing mean income scores between the *gender diverse*** **and *cisgender* group parents.**

*In the exclusions section, it would seem that after these exclusions were made, the total number of participants listed in the Participants section remained. However, given the placement of this, it was not clear (until the end of the section) whether these were exclusions beyond the N=145. Please start by listing the total number of participants run; that is: We started with a total of X participants; however, “during data collection, we noticed…” to clarify.*

**Thank you for this suggestion. We have restructured the Participants section slightly so that we include exclusion-related information for each subgroup of participants in our study (gender diverse adolescents and their parents, and cisgender adolescents and their parents). For each subgroup, we describe how many responses we received, how many we excluded for specific reasons, and how many participants were left in the final sample.**

*Please provide a table of correlations that corresponds to the regression analyses.*

**We were not exactly sure what table of correlations you are seeking, but we do now report a table of the Pearson’s *r* correlations between the three main stereotype measures (adolescent self-report, parent self-report, and adolescent prediction about the caregiver) in Supplementary Material 7. Apologies if this is not what you were seeking.**

**If you were seeking correlations between our categorical predictors (gender diverse vs. cisgender, parent vs. adolescent), the analogous information is captured in the regression summarized in Table 6, which examines group differences in self-reported stereotyping across in both adolescents and parents in the gender diverse and cisgender groups.**

**Similarly, if you were seeking information about how the relationship between parents’ and adolescents’ stereotyping might be different in the gender diverse vs. cisgender dyads, this information is captured in Tables S5 and S6 in the Supplementary Materials.**

**If you meant to suggest a different set of correlations, please let us know and we would be happy to add it.**

*Stylistically, I would recommend removing the ‘road map’ on page 4 and instead relying on your headers to guide the reader.*

**Thank you for this suggestion; we have removed the road map text and instead use conceptual headers throughout.**

*On page 7 you seem to suggest that an advantage of this measure is that it has “often produced clear indications of gender stereotyping in this age group”. I would rephrase as it is not clear from this wording why this is an advantage. I see this instead as a previous finding that is relevant to your research questions and predictions.*

**Thank you for this point. We have removed the text in this paragraph that lists this as an advantage of the measure. Instead, we highlight the results observed in past studies using the trait subscale of the OAT-AM.**

*I was not clear about the dfs provided for the F-statistic in the first paragraph of page 18. Also, you note that this compares boys, girls, and non-binary/other adolescents, but in the discussion, you note findings regarding boys and girls. If this is an omnibus F with three groups please provide post-hoc direct comparisons.*

**Thank you for raising this point. The F statistic refers to a one-way ANOVA comparing mean stereotyping scores among boys, girls, and nonbinary/other adolescents; thus, there were three groups, and 2 degrees of freedom. In addition, as you suggested, we have added post-hoc pairwise tests as well, which match the finding pointed out in the discussion.**

*To decrease possible confusion, I would recommend referring to tables in the supplement as Table S1, etc.*

**Thank you, we have adjusted how we refer to tables in the supplement.**

*Although you list all ten traits in the supplement, I think that it would also be helpful for readers to see these listed in the manuscript on page 13.*

**Thank you. We now include a table with all the items (including their means, standard errors, and the number of participants who skipped each) in the “Gender stereotype endorsement” subsection of the Results section (Table 3).**

*On page 14, you note that more people skipped specific items; you might include those ns in Table S7.*

**Thank you for this suggestion - we have added participant N’s for those who skipped each item in the table mentioned above (Table 3 in the manuscript).**
